# Prognostic Factors Associated with Treatment Outcomes in Endodontic/Periodontal Lesions: A Systematic Literature Review

**DOI:** 10.3390/diagnostics16213686

**Published:** 2026-11-08

**Authors:** Ana Kostenkova, Greta Lodienė, Uršulina Lyndo, Eglė Zasčiurinskienė

**Affiliations:** 1 Faculty of Odontology, Lithuanian University of Health Sciences, LT-50106 Kaunas, Lithuania; 2 Department of Dental and Oral Pathology, Lithuanian University of Health Sciences, LT-50106 Kaunas, Lithuania; 3 Department of Orthodontics, Lithuanian University of Health Sciences, LT-50106 Kaunas, Lithuania

**Keywords:** endodontic/periodontal lesion, treatment prognosis, prognostic factors, endodontic outcome, periodontal outcome

## Abstract

**Background/Objectives:** The complex nature of endodontic/periodontal lesions complicates treatment prognosis, making long-term tooth survival challenging to predict. Identifying key prognostic factors can facilitate evidence-based clinical decision-making. This systematic review aims to evaluate the available evidence on factors associated with treatment outcomes in endodontic/periodontal lesions and to identify potential prognostic indicators. **Methods:** PubMed/MEDLINE, Embase, Web of Science and Cochrane Library were searched from 1 June to 1 July 2026 with no restrictions. Observational studies investigating associations between baseline prognostic factors and treatment outcomes in adults with endodontic/periodontal lesions were eligible for inclusion. Study selection and data extraction were performed independently by two reviewers. Risk of bias was assessed using the Quality In Prognosis Studies (QUIPS) tool, and certainty of evidence was evaluated using GRADE. Due to substantial methodological heterogeneity, the findings were synthesized narratively rather than through meta-analysis. **Results:** Four retrospective studies comprising 501 teeth were included. Risk of bias was predominantly moderate to high, and certainty of evidence was low. No single factor demonstrated directionally consistent independent association across the available adjusted analyses. **Conclusions:** Despite limited evidence, an overall pattern suggests that endodontic/periodontal lesion prognosis may be most frequently associated with the extent of periodontal tissue destruction and the amount of remaining periodontal support. Prospective studies using standardized endodontic/periodontal lesion definitions, outcomes and appropriately adjusted multivariable prognostic models are needed.

## 1. Introduction

An endodontic/periodontal lesion (EPL) is a pathological condition that develops as a result of bacterial infection affecting both the pulp and the periodontal tissues through their anatomical interconnections. The primary aetiological factor is microbial infection, which induces periodontal inflammation, leading to destruction of the junctional epithelium and progressive attachment loss. As periodontal breakdown progresses, increased probing depth (PD) and clinical attachment loss (CAL) may permit bacterial migration to the pulp via lateral canals or the apical foramen. Conversely, pulpal necrosis resulting from caries, trauma, or restorative procedures can cause apical periodontitis, with infection spreading coronally along the periodontal ligament [1,2,3,4].

EPLs pose significant diagnostic and therapeutic challenges. Their management requires a multidisciplinary approach. The combined nature of these pathologies complicates prognosis, as the healing and long-term tooth survival remain difficult to predict. Patients with EPL frequently seek professional guidance regarding the relative merits of treatment versus extraction. Such discussions often become complex, as clinicians may exhibit hesitance in providing definitive prognostic statements due to the unpredictability associated with treatment outcomes [2]. Although EPL treatment protocols are frequently complex, staged, and both technically and financially demanding [2], tooth extraction followed by implant placement is associated with inherent biological limitations, particularly in patients with EPL, given the increased prevalence of peri-implant diseases among individuals with a history of periodontal disease [5], underscoring the importance of preserving natural dentition whenever feasible.

While prognostic factors for periodontal and endodontic treatment outcomes have been studied separately [6,7], strong evidence on the prognosis of EPL treatment is lacking. Recent systematic evidence indicates that periodontitis may be associated with subclinical histopathological changes in the dental pulp, further complicating diagnosis of combined endodontic/periodontal involvement [8]. According to the most recent classification [9], prognosis is predominantly influenced by the presence or absence of root damage and/or periodontitis, which in turn depends on the extent of periodontal destruction and the anatomical characteristics of the affected tooth. However, these criteria are excessively broad to enable reliable clinical decision-making. Although contemporary clinical guidance has proposed specific criteria for tooth extraction in EPLs, including severe loss of periodontal support, grade III mobility and untreatable structural root damage, these recommendations are largely consensus-based and are not supported by direct prognostic studies [10]. By delineating the prognostic factors with precision, clinicians would be equipped to enhance evidence-based decision-making and improve communication with patients regarding expected treatment outcomes. This review seeks to identify and critically assess the prognostic factors that affect the outcomes of EPL treatment.

## 2. Materials and Methods

### 2.1. Protocol and Registration

The conduct and reporting of this systematic review was guided by the Cochrane Handbook [11] and the PRISMA (Preferred Reporting Items for Systematic reviews and Meta-Analyses) statement [12]. This review was registered in an international prospective register of systematic reviews (PROSPERO) under the number CRD420251102815.

### 2.2. Focus Question

Using the Participants–Intervention–Comparison–Outcome–Study design (PICOS) framework [13], we included observational studies (S) and formulated the PICO question: In adult patients with treated endodontic/periodontal lesions (P), are any patient baseline characteristics (I), compared with their absence or with lower or reference levels of the same characteristics (C), associated with treatment response, treatment success or failure, or tooth survival (O)?

### 2.3. Eligibility Criteria and Search Strategy

A systematic search for relevant articles was conducted in the PubMed/MEDLINE, Embase, Web of Science and Cochrane Library databases. The electronic search was conducted between 1 June and 1 July 2026 and covered all records indexed from database inception to the date of the final search. No restrictions regarding publication date, language, or study design were applied. A comprehensive search of scientific electronic data was conducted independently by two investigators (AK, UL). The complete set of keywords and their combinations with Boolean operators used for the search across each of the selected databases is presented in Supplementary Table S1.

Two investigators (AK, EZ) independently analysed the titles and abstracts of the studies that met the eligibility criteria. Duplicate records were identified and removed utilising Mendeley reference management software (Version 2.149.0, Elsevier, Amsterdam, The Netherlands), and the remaining articles were screened according to the inclusion and exclusion criteria outlined in Table 1.

### 2.4. Risk of Bias and Certainty Assessment

Risk of bias was assessed using the Quality In Prognosis Studies (QUIPS) tool, covering six domains: study participation, study attrition, prognostic factor measurement, outcome measurement, study confounding, and statistical analysis and reporting. Attention was paid to the adequacy of adjustment for relevant confounders, including baseline periodontal severity, patient-related characteristics, tooth-related factors and treatment characteristics. Statistical analysis and reporting were evaluated with consideration of the appropriateness of the analytical methods, multivariable adjustment, model development, and completeness of effect estimate reporting. Each domain was judged as having low, moderate, or high risk of bias, with disagreements resolved by a third author [14]. The certainty of evidence for each prognostic factor was assessed using the GRADE approach, considering risk of bias, inconsistency, indirectness, imprecision, and publication bias [15].

### 2.5. Data Extraction

Two reviewers (AK, GL) independently extracted study design, participant and tooth characteristics, EPL definitions, treatment protocols, follow-up duration, outcome definitions, candidate prognostic factors, factor measurement methods, statistical analyses, and reported prognostic associations. Where both univariable and multivariable analyses were reported, adjusted estimates were prioritised in the interpretation of prognostic associations. Unadjusted associations were considered supportive but were not interpreted as evidence of independent prognostic value. Owing to substantial heterogeneity in outcome definitions, factor categorisation, follow-up periods, and statistical effect measures, quantitative pooling was considered inappropriate and the findings were synthesised narratively according to the direction and consistency of associations. Disagreements were resolved through discussion with other reviewers.

## 3. Results

### 3.1. Selected Studies

**Figure 1:**
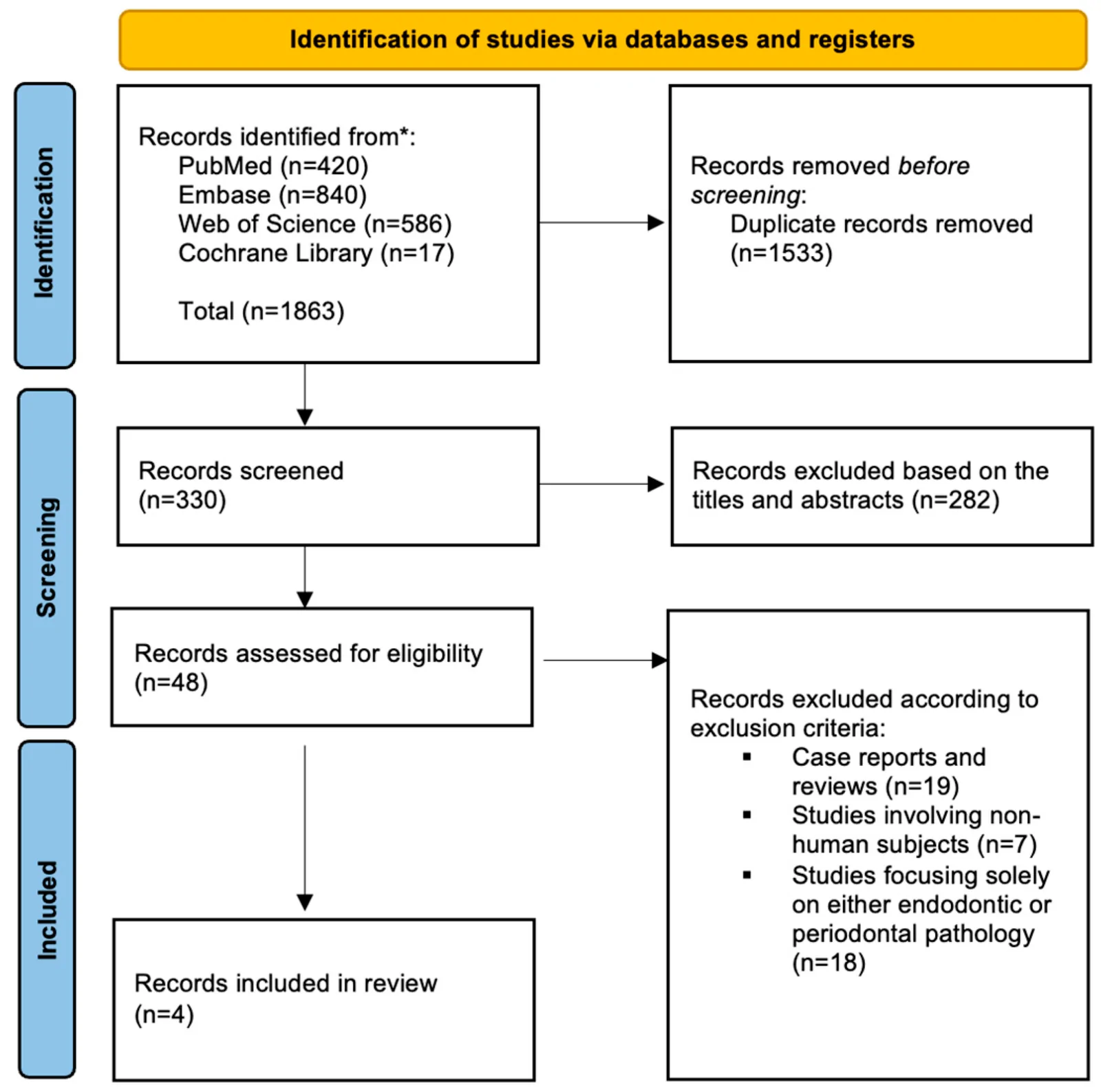
PRISMA flowchart.

The selection process of the studies is outlined in the PRISMA flowchart (Figure 1). Following the electronic search and the removal of duplicates, 330 records underwent title and abstract screening, of which 48 reports were assessed in full text. After a comprehensive screening process, 44 records were excluded based on the established exclusion criteria (Table 1). Finally, 4 studies were included in this review.

### 3.2. Quality Assessment

Overall, the included studies [16,17,18,19] demonstrated a predominantly moderate-to-high risk of bias (Table 2). Study participation was rated as having a high risk of bias in two studies [17,19], primarily due to retrospective case selection, incomplete clinical records and restrictive eligibility criteria. Outcome measurement raised additional concerns in studies using composite treatment response definitions that incorporated clinical parameters also evaluated as prognostic factors [16,17,18]. Confounding and statistical analysis represented major sources of potential bias. Guo et al. [17] relied on unadjusted comparisons without accounting for potential confounders, while Wong et al. [18] excluded candidate confounders from the final regression analyses based on statistical significance.

The most frequent concerns were retrospective inclusion of cases with complete clinical records or adequate follow-up, which may have introduced selection bias. Limitations in statistical analysis and reporting were also identified, particularly in Guo et al. [17], which lacked multivariable prognostic modelling. In contrast, prognostic factor measurement was generally more robust, with clearly defined clinical and radiographic parameters.

The certainty of evidence was further limited by the small number of available studies, heterogeneous outcome definitions, and imprecision of all prognostic estimates, and was judged as low.

### 3.3. Methodological Characteristics of the Included Studies

The general characteristics of each study are summarised in Table 3. All participants across the included studies were adults aged 18 years and older diagnosed with EPL. Follow-up periods varied considerably, ranging from six months [16,17] to at least one year [18,19], whereas Kimura et al. [19] evaluated long-term tooth survival using time-to-event analysis.

All studies collected demographic and core clinical or radiographic variables, although the range of investigated prognostic factors differed (Table 3). Considerable heterogeneity was observed in treatment outcome definitions across studies (Table 4).

Fan et al. [16] analysed 140 teeth from 140 individuals, so each participant contributed one tooth and within-patient clustering was not present. Guo et al. [17] included 86 teeth from either 72 cases; therefore, some patients contributed more than one tooth. Wong et al. [18] and Kimura et al. [19] included 88 teeth and 187 teeth respectively, but the number of unique patients was not clearly reported.

### 3.4. Prognostic Factors Associated with Treatment Outcomes in EPLs

In the study conducted by Wong et al. [18], the favourable outcome rate of EPL treatment was reported to be 46.6%. The predominant cause of treatment failure was identified as tooth mobility (53.6%), followed by other reasons, pain and vertical root fracture. Fan et al. [16] reported 51.4% high responders, whereas Guo et al. [17] classified 67.4% of teeth as having an effective treatment outcome. Kimura et al. [19] extended the available evidence by evaluating long-term tooth survival. The estimated 1- and 5-year survival rates were 83.8% and 58.9%, respectively.

The results of the evidence synthesis are summarised in Table 5. Given the heterogeneity in the terminology and prognostic variables assessed across the included studies, individual factors were additionally grouped into broader clinically related domains to facilitate cross-study interpretation in this review (Table 5). The most consistent adverse prognostic associations clustered within the domain of periodontal destruction and remaining support, particularly for PD, CAL, radiographic bone loss and CR. In contrast, host-, anatomical- and endodontic-related factors demonstrated more variable study-specific associations.

### 3.5. Meta-Analysis

Owing to substantial heterogeneity in outcome definitions, follow-up periods, prognostic factor measurement and statistical effect measures, a quantitative meta-analysis was considered inappropriate.

## 4. Discussion

Clinical decision-making regarding tooth retention versus extraction is complex and multifactorial, requiring thorough evaluation of biological, functional and patient-related factors. The identification of teeth with a favourable prognosis could contribute to a reduction in unnecessary extractions, ensuring that patients receive care that is biologically, economically, and ethically sound. To our knowledge, this is the first systematic review specifically focused on baseline prognostic factors associated with treatment outcomes and tooth survival in EPLs.

This review reveals that the incidence of treatment failure cases varies between 31.8% and 48.57% [16,18]. Additionally, Kimura et al.’s [19] analysis showed a substantial reduction in estimated survival during longer-term follow-up. No prognostic factor demonstrated a stable and directionally consistent independent association across all available adjusted analyses, emphasising the need for cautious interpretation and suggesting that methodological heterogeneity, differences in EPL characteristics, treatment protocols, and outcome definitions may partly explain these inconsistent findings.

Firstly, from a periodontal perspective, tooth survival and treatment success should not be regarded as interchangeable outcomes. A tooth may remain in situ despite persistent deep probing depths, bleeding or suppuration, mobility, and incomplete attachment or radiographic healing. Interpretation of the present findings is also constrained by changes in EPL classification. The classic classification proposed by Simon et al. [20] categorised EPL according to the presumed primary pathway of disease. In contrast, the 2017 World Workshop classification [9] shifted the emphasis from retrospective attribution of disease origin to clinical features with direct implications for prognosis and treatment, particularly the presence of root damage, the presence of periodontitis, and the extent of periodontal pocketing. The differences in used classifications are an additional source of clinical heterogeneity. These differences may have resulted in study populations with varying baseline disease severity, potentially influencing treatment outcomes and the observed prognostic associations. Although Wong et al. [18] discussed Simon et al.’s classification, none of the included studies provided outcome data stratified according to these categories. Consequently, it was not possible to determine whether the primary origin of the lesion influenced treatment prognosis. This distinction may be clinically relevant, as lesions with predominantly endodontic involvement may respond differently to treatment than lesions associated with extensive periodontal destruction. The lack of consistent classification and subgroup-specific outcomes therefore limits the comparability and generalisability of the findings.

Although no single variable demonstrated a consistent association with treatment outcome across all the included studies, the overall findings suggest that periodontal tissue destruction and remaining periodontal support represent central prognostic considerations in EPLs. The primary prognostic factors associated with EPLs appear to be predominantly periodontal in nature and exhibit a close interrelationship. However, probing depth (PD), clinical attachment loss (CAL), radiographic bone loss, and tooth mobility should not necessarily be interpreted as independent prognostic factors, as they may reflect overlapping manifestations of underlying periodontal disease severity. This distinction is clinically relevant because several commonly investigated variables may not represent independent prognostic domains, but overlapping expressions of the same underlying periodontal disease burden. Two studies of this review [16,17] have identified the loss of clinical attachment as a significant prognostic factor, which is directly correlated with the development of periodontal pockets and subsequent alveolar bone loss. The study conducted by Fan et al. [16] reported no significant differences between horizontal and vertical bone resorption types concerning the prognosis of EPLs. Notably, bone loss extending into the apical third of the root was considered a marker of poor prognosis [18]. This condition facilitates deeper microbial infiltration into periodontal tissues, rendering infection more resistant to treatment and yielding less predictable healing outcomes. As periodontal bone support is lost, the clinical crown-to-root ratio becomes increasingly unfavourable, potentially compromising biomechanical stability and long-term tooth retention. This deficiency increases the risk of tooth mobility, root fracture, or further attachment loss. Importantly, even if the endodontic infection is successfully eliminated, an unfavourable crown/root ratio limits the long-term survival of the affected tooth [21]. The recurrent identification of these support-related variables across studies raises the hypothesis that the overall severity of periodontal support loss may be more prognostically relevant than any single periodontal measurement considered in isolation.

Interestingly, bleeding on probing, assessed through the sulcus bleeding index (SBI), was identified as a significant prognostic in only one study [17]. This finding is somewhat unexpected, as bleeding on probing represents one of the primary clinical signs of periodontal inflammation and is routinely used to assess periodontal disease activity [22]. However, its lack of prognostic significance in EPLs may be explained by the fact that bleeding on probing is highly dynamic and can improve rapidly following initial therapy and improved plaque control. Due to the retrospective design of the study, bleeding on probing may not have been consistently or accurately documented, potentially limiting its ability to demonstrate a significant influence on treatment outcomes.

Tooth mobility showed inconsistent prognostic findings. It may be an important factor, as it signifies both alveolar bone loss and damage to the periodontal ligament, which collectively compromise tooth stability. However, current review evidence does not establish mobility as an independent prognostic factor once other measures of periodontal destruction are considered. It is noteworthy that gingival recession has not been extensively studied as a prognostic factor, despite its potential clinical significance. Increased root exposure to the oral environment facilitates microbial colonisation of root surfaces and may establish a pathway for microorganisms to penetrate the pulp through exposed dentinal tubules [23]. Histological studies have identified more pronounced pulpal changes in teeth exhibiting gingival recession, suggesting that pulpal health may be compromised [24].

Regarding periodontal factors, occlusal trauma and periodontal defect morphology have not been evaluated as independent predictors of treatment outcome in longitudinal EPL studies and warrant future investigation. The existing literature indicates that traumatic occlusion significantly impacts periodontal tissues, resulting in occlusal trauma and associated periodontal changes [25]. However, there is a notable deficiency of studies investigating the effects of occlusal trauma on the dental pulp. While excessive occlusal forces do not initiate periodontal disease, they may exacerbate pre-existing inflammation by increasing tooth mobility, widening the periodontal ligament space, and accelerating alveolar bone loss by the presence of vertical bone defects. In teeth that are already compromised by periodontal disease, such biomechanical stresses may promote the apical spread of plaque-induced inflammation in the pocket and contribute to the formation of vertical bone defects [25]. Specifically, the presence of infrabony defects has been linked to more advanced periodontal breakdown and serves as a predictor of further bone loss, which may ultimately lead to tooth loss [26]. In the context of EPLs, infrabony defects may facilitate communication between the periodontal and pulp tissues, thereby influencing the complexity of lesions and treatment response due to greater attachment loss, compromised periodontal support, and increased susceptibility to disease progression. Furthermore, occlusal trauma may impede post-treatment recovery following endodontic/periodontal therapy, directly affecting prognostic outcomes.

Concerning endodontic variables, the prognostic relevance of periapical status was inconsistent. Guo et al. [17] reported higher PAI values in ineffective cases. Kimura et al. [19] further demonstrated that the radiographic morphology of EPLs may carry prognostic relevance, with J- and U-shaped lesions being associated with an increased risk of tooth loss. These lesion configurations may reflect a more extensive continuity of osseous destruction along the root surface and periapical region, potentially indicating greater loss of periodontal support and a more complex interaction between endodontic and periodontal disease. In contrast, PAI was not retained in Fan et al.’s [16] multivariable model, while Wong et al. [18] found no significant association with PARL.

Additionally, two studies [16,17] have identified the number of root canals as a significant prognostic factor. Teeth with a greater number of canals are more susceptible to missed or inadequately treated canals and residual infection, which can hinder healing and negatively impact the overall prognosis [27]. This consideration is especially pertinent in the context of the complexity of infection dissemination within multi-rooted teeth [28]. Infection may be confined to a single canal, resulting in false-positive results in diagnostic pulp sensitivity tests, which can mislead clinicians. A study conducted by Ruetters et al. [29] investigated the effect of endodontic treatment alone in patients diagnosed with periodontitis. The majority of successful outcomes were observed in single-rooted teeth, while treatment failures were exclusively associated with multi-rooted teeth. Interestingly, the presence of caries, and the type and complexity of restorations were not identified as significant prognostic factors in the reviewed studies. Evidence suggests that these variables are more closely linked to the aetiology and prevalence of EPLs, while the success of treatment primarily depends on the effective elimination of bacteria from both the endodontic and periodontal spaces [30].

There is limited evidence on how conditions such as diabetes and systemic bone diseases impact the prognosis. Smoking history was identified as a significant factor in one study [16]. This finding aligns with extensive evidence demonstrating that smoking adversely affects both periodontal [31,32,33] and endodontic prognosis [34,35]. Smoking impairs periodontal healing by altering immune responses, restricting blood flow through nicotine-induced vasoconstriction, disrupting fibroblast and osteoblast activity, and modifying the oral microbiota [36,37,38].

Existing research regarding prognostic factors in patients undergoing treatment for periodontitis [6,39,40] indicates that elements such as stress, tooth position, dental trauma history, iatrogenic complications, sinus tract presence and type of bone defect may impact treatment outcomes. Furthermore, patient preferences regarding tooth retention or extraction may have influenced reported survival outcomes independently of clinical prognosis. However, these factors have not yet been assessed within the specific context of EPLs, thereby indicating critical gaps for future research.

Certain methodological characteristics of the included studies warrant acknowledgement, as they may have influenced the results. Firstly, the time frame of the included records (from 2008 onward) may limit the generalisability of the findings, given that treatment protocols and clinical techniques have evolved over time, potentially affecting both treatment efficacy and prognosis. Furthermore, the retrospective design of most studies inherently restricts the standardisation or control of treatment protocols, which may introduce variability in outcomes and confound the assessment of prognostic factors. One of the main limitations is that the criteria used to evaluate EPL prognosis varied substantially among the included studies (Table 4). The variability in outcome definitions and assessment criteria limits direct comparison between studies and may partly explain the differences in reported prognostic factors. The types and definitions of EPL also differed among the included studies, reflecting the use of different classification systems and inclusion criteria. Furthermore, follow-up duration varied considerably between studies, which may have influenced reported outcomes, as short-term tooth retention or clinical improvement does not necessarily reflect long-term survival [19].

## 5. Conclusions

The certainty of the available evidence is low due to the small number of studies, retrospective designs, heterogeneous outcome definitions and follow-up periods. Evidence is insufficient to validate EPL grade as a stand-alone prognostic system or to establish the independent prognostic value of individual periodontal parameters. Although no single prognostic factor showed a consistent independent association across all studies, an overall pattern suggests a hypothesis that EPL prognosis may be most frequently associated with the extent of periodontal tissue destruction and the amount of remaining periodontal support. These findings suggest that the periodontal component represents a central prognostic domain in treated EPLs, while patient-level, endodontic, and treatment-related factors may act as additional modifiers. Several periodontal variables may reflect partially overlapping manifestations of the same underlying disease burden; however, this concept requires confirmation in prospective studies using standardized outcome definitions and appropriately adjusted multivariable prognostic models.

## Figures and Tables

**Table 1:** Inclusion and exclusion criteria used to qualify studies for review.

Inclusion Criteria	Exclusion Criteria
Observational studies involving adult patients with at least one tooth with a confirmed diagnosis of an EPL Observational studies evaluating the association between at least one baseline patient-, tooth-, periodontal-, endodontic-, or radiographic-related factor and a subsequent EPL treatment outcome or tooth survival	Studies focusing solely on either endodontic or periodontal pathology without addressing the combined nature of the lesion Non-human studies Studies lacking documentation of pre-treatment clinical parameters Studies involving teeth where surgical endodontic treatment was performed Case reports, review articles or meta-analyses

**Table 2:** Risk of bias assessment according to the Quality In Prognosis Studies (QUIPS) tool.

Study	Participation	Attrition	Prognostic Factor Measurement	Outcome Measurement	Confounding	Statistical Analysis and Reporting	Overall Risk of Bias
Fan et al., 2020 [16]	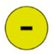	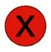	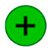	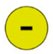	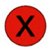	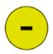	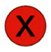
Guo et al., 2022 [17]	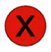	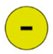	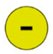	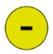	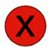	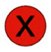	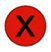
Wong et al., 2024 [18]	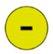	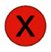	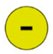	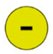	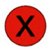	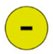	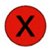
Kimura et al., 2026 [19]	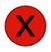	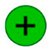	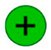	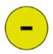	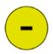	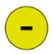	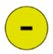

Green “+” = low; yellow “−” = moderate; red “X” = high.

**Table 3:** General characteristics of the included studies.

Study	Country	Study Design	Record Retrieval Timeframe	Sample Size (Number of Participants; Teeth)	Follow-Up	EPL Classification	Treatment	Prognostic Factors Evaluated	Reported Outcome Associations
Wong et al., 2024 [18]	Australia	Retrospective observational study	2008–2021	140 patients; 88 teeth	≥1 year after endodontic and/or periodontal treatment	All types of EPL, no classification specified	Only endodontic Only periodontal Combined treatment	Demographic variables Endodontic-related variables Periodontal-related variables Treatment variables	Bone loss to the apical third, deep probing depths, and a history of periodontal disease
Guo et al., 2022 [17]	China	Retrospective observational study	2018–2021	74 patients; 86 teeth	1, 3, 6 months after combined treatment	Combined EPL	Combined non-surgical endodontic and periodontal treatment	Demographic variables Clinical parameters	Patient’s oral hygiene maintenance, the loss of attachment, the degree of loosening, the clinical crown-to-root ratio, the periapical index, and the number of root canals
Fan et al., 2020 [16]	China	Retrospective observational study	2016–2018	Number of patients not reported; 140 teeth	≥6 months after periodontal therapy	Grade 2–3 EPL (2017 World Workshop classification)	Combined non-surgical endodontic and periodontal treatment	Demographic data Clinical parameters	High PD and CAL, number of root canals, smoking, and serious full-mouth periodontitis
Kimura et al., 2026 [19]	Japan	Retrospective cohort study	April 2015–March 2021	Number of patients not reported; 187 teeth	≥6 months after treatment	Grade 1–3 EPL (2017 World Workshop classification)	Endodontic treatment followed by periodontal therapy, periodontal surgery when indicated	Demographic data Clinical parameters Radiographic parameters	Age, EPL grade, PD, bone loss, I-J-U radiographic subclassification

PD—probing depth, CAL—clinical attachment loss, PAI—periapical index, EPL—endodontic/periodontal lesion, AAP—American Academy of Periodontology.

**Table 4:** Study-specific definitions of treatment outcomes in the included studies.

Wong et al., 2024 [18]	Success (46.6%) PD ≤ 4 mm BoP absent TM absent Radiographic evidence healing of periapical tissues or absence of periapical lesion Asymptomatic		Survival (21.6%) PD > 4 mm BoP present TM present Radiographic evidence of incomplete healing of periapical tissues Re-infection of the canal space	Failure (31.8%) Extraction of the tooth
Guo et al., 2022 [17]	Cured PD < 3 mm SBI < 1 CAL stable TM decrease No radiolucency in the apical area, normal alveolar bone density Asymptomatic Improved chewing function No gingival swelling	Improved PD < 4 mm SBI < 2 CAL stable TM decrease PAI was decreased and less than or equal to 1 Asymptomatic Improved chewing function Mild inflammation of the affected teeth and gingiva	Ineffective (32.56%) PD > 5 mm SBI > 3 CAL increased TM increased PAI presence Unchanged or enlargement of the sinus or fistula hole Positive percussion test Conscious symptoms Disturbed chewing function Red and swollen gums	
Fan et al., 2020 [16]	High responder group (51.43%) PD ≤ 5 mm or ≥2 mm improvement in PD SBI ≤ 2 CAL stable or decreased TM stable or decreased PAI no increase Asymptomatic Improved chewing function No gingival swelling No sinus tract No pain		Low responder group (48.57%) PD > 5 mm or less than 2 mm improvement in PD SBI ≥ 3 CAL increased TM increased PAI increased Impaired chewing function Redness and swollen gums Sinus tract present	
Kimura et al., 2026 [19]	Tooth survival Tooth remained in situ until the last observation or end of follow-up		Tooth extraction Time from root canal filling to extraction	
	Kimura et al. evaluated time to tooth extraction and did not distinguish complete healing from incomplete healing among retained teeth. Tooth retention should therefore not be interpreted as equivalent to treatment success.			

BoP—bleeding on probing, PD—probing depth, SBI—sulcus bleeding index, TM—tooth mobility, PAI—periapical index, CAL—clinical attachment loss.

**Table 5:** Domain-based synthesis of prognostic factors investigated across the included studies.

Prognostic Variable/Study	Fan et al., 2020 [16] Clinical Response Evaluation	Guo et al., 2022 [17] Treatment Effectiveness Evaluation	Wong et al., 2024 [18] Treatment Success/Survival/Failure Evaluation	Kimura et al., 2026 [19] Tooth Survival Evaluation	Certainty of Evidence (GRADE) [15]	Clinical Domain
Age	◄► U: *p* = 0.991	◄► U	◄► R: OR 0.998 (95% CI 0.970–1.028), *p* = 0.908	▼ A: HR 1.04 per year (1.01–1.06), *p* = 0.002	Low	Patient context
Gender	◄► U: *p* = 0.657	Not evaluated	◄► R: OR 1.150 (0.526–2.514), *p* = 0.727	◄► A: HR 1.60 (0.94–2.74), *p* = 0.084	Low	
Diabetes/systemic bone disease	Not evaluated	Not evaluated	Diabetes ◄► R: OR 0.332, *p* = 0.082; Systemic bone disease ◄► R: OR 2.846, *p* = 0.094	Not evaluated	Low	
Smoking history	▼ A: OR 5.18 (1.14–23.63), *p* = 0.034	Not evaluated	◄► R: OR 1.134, *p* = 0.792	Not evaluated	Low	
Medications	Not evaluated	Not evaluated	◄► R: OR 0.717 (0.303–1.699), *p* = 0.450	Not evaluated	Low	
Tooth type	Not evaluated	Not evaluated	Not evaluated	◄► A: HR 1.15 (0.62–2.13), *p* = 0.65	Low	Tooth anatomy and complexity
Number of canals	▼ A: OR 2.91 (1.22–6.94), *p* = 0.016	▼ U: t = 3.65, *p* < 0.05;	◄► R: *p* ≥ 0.328	Not evaluated	Low	
Treatment-related factors	Not evaluated	Not evaluated	◄► R	Not evaluated	Low	Treatment-related factors
Restoration	Not evaluated	Not evaluated	◄► R: OR 0.434, *p* = 0.088; absence of a crown: R: OR 0.307, *p* = 0.096	Not evaluated	Low	
Presence of iatrogenic dental complications	Not evaluated	Not evaluated	◄► R: OR 1.227 (0.514–2.930), *p* = 0.645	Not evaluated	Low	
Periapical status	▼ U: *p* = 0.002	▼ U: *p* < 0.05	Not evaluated	◄► A: HR 0.96, *p* = 0.91	Low	Endodontic factors
Presence of previous endodontic treatment	Not evaluated	Not evaluated	◄► R: OR 0.807 (0.124–5.248), *p* = 0.822	◄► U: *p* = 0.30	Low	
Clinical crown/root ratio	▼ U: *p* = 0.022	▼ U: *p* < 0.05;	Not evaluated	Not evaluated	Low	Periodontal destruction and remaining support
Tooth mobility	▼ U: *p* = 0.004,	▼ U: *p* < 0.05;	◄► R: OR 1.565, *p* = 0.513	◄► A: HR 1.41, *p* = 0.24	Low	
History of periodontal disease	Not evaluated	Not evaluated	▼ R: OR 7.705 (1.603–37.037), *p* = 0.011 (higher odds of a favourable outcome without previous periodontal disease)	Not evaluated	Low	
Bone loss	◄► U: *p* = 0.089	Not evaluated	▼ R: OR 0.300 (0.104–0.866), *p* = 0.026 (lower odds of a favourable outcome with bone loss extending to the apical third)	▼ A: HR 1.02 per 1% increase (1.01–1.03), *p* = 0.005	Low	
PD	▼ A: OR 2.41 (1.24–4.65), *p* = 0.009	◄►	▼ R: PD 5–7 mm, OR 0.147, *p* = 0.010; PD 8–10 mm, OR 0.126, *p* = 0.005; >10 mm nonsignificant	▼ A: HR 1.17 per mm (1.03–1.32), *p* = 0.009	Low	
CAL	▼ A: OR 2.34 (1.30–4.22), *p* = 0.005	▼ U: *p* < 0.05	Not evaluated	Not evaluated	Low	
Furcation involvement	Not evaluated	Not evaluated	◄► R: OR 0.456 (0.160–1.301), *p* = 0.142	◄► U: *p* = 0.96	Low	
Bleeding on probing	◄►U: *p* = 0.080	▼ U: *p* < 0.05	◄► R: OR 0.617, *p* = 0.475	Not evaluated	Low	
Patient’s oral hygiene	◄► U: *p* = 0.620	Not evaluated	◄► R: *p* ≥ 0.298	Not evaluated	Low	
Periodontitis stage and grade	▼ A: OR 2.84 (1.07–7.52), *p* = 0.036	Not evaluated	◄► R: OR 0.470, *p* = 0.225	◄► A: HR 0.68, *p* = 0.61	Low	
EPL grade	Not evaluated	Not evaluated	Not evaluated	▼ A: 1, HR 3.74 (1.14–12.28), *p* = 0.030 (higher hazard of tooth extraction in Grade 2–3 EPLs)	Low	
I-J-U radiographic subclassification	Not evaluated	Not evaluated	Not evaluated	▼ A: J-shaped HR 4.54 (1.00–20.60), *p* = 0.050; U-shaped HR 29.10 (5.48–154.67), *p* < 0.001 versus I-shaped (higher hazard of tooth extraction, particularly with U-shaped lesions)	Low	

A, multivariable-adjusted effect estimate; U, unadjusted comparison or log-rank test; R, reported ordinal logistic regression association without documented adequate multivariable confounder adjustment, ▼ statistically significant adverse association; ◄► no statistically significant association; “Not evaluated” indicates that the factor was not analysed prognostically.

## Data Availability

All data supporting the findings of this systematic review were obtained from the published studies included in the review and are cited in the article. No new primary datasets were generated or deposited in a public repository.

## Supplementary Materials

The following supporting information can be downloaded at https://www.mdpi.com/article/10.3390/diagnostics16213686/s1, Table S1: The search strategy. File S1: PRISMA Checklist.
