# Supplementary material for: Prognostic Factors Associated with Treatment Outcomes in Endodontic/Periodontal Lesions: A Systematic Literature Review
Source: Diagnostics (Basel). 2026 Nov 8;16(21):3686. doi: 10.3390/diagnostics16213686 (PMC13762132; doi:10.3390/diagnostics16213686)
Supplement: Supplementary file 1 [file diagnostics-16-03686-s001.zip › diagnostics-4674960-supplementary.pdf]

Table S1. Database-specific electronic search strategies.

Database	Search strategy (keywords, controlled vocabulary, and Boolean operators)	Records identified (n)
**PubMed/MEDLINE**	(<br>"endodontic-periodontal"[tiab] OR "endodontic periodontal"[tiab] OR "periodontal-endodontic"[tiab] OR "endo-perio"[tiab] OR endoperiodontal[tiab] OR "combined endodontic and periodontal"[tiab] OR "combined periodontal and endodontic"[tiab] OR<br>(<br>("Periodontitis"[MeSH Terms] OR periodontitis[tiab] OR "periodontal disease"[tiab] OR "periodontal diseases"[tiab] OR "periodontal lesion"[tiab])<br>AND<br>("Dental Pulp Diseases"[MeSH Terms] OR "Endodontics"[MeSH Terms] OR "Root Canal Therapy"[MeSH Terms] OR endodontic*[tiab] OR "pulp necrosis"[tiab] OR "pulp disease"[tiab] OR "root canal"[tiab])<br>)<br>)<br>AND<br>(<br>"Prognosis"[MeSH Terms] OR "Treatment Outcome"[MeSH Terms] OR "Tooth Loss"[MeSH Terms] OR prognos*[tiab] OR predict*[tiab] OR "risk factor"[tiab] OR "risk factors"[tiab] OR outcome*[tiab] OR success*[tiab] OR failure*[tiab] OR surviv*[tiab] OR "tooth retention"[tiab] OR extract*[tiab] OR "treatment response"[tiab]<br>)	420
**Embase**	(<br>(<br>('endodontic-periodontal' OR 'endodontic periodontal' OR 'periodontal-endodontic' OR 'endo-perio' OR endoperiodontal OR 'combined endodontic and periodontal' OR 'combined periodontal and endodontic'):ti,ab,kw<br>)<br>OR<br>(<br>('periodontitis'/exp OR (periodontitis OR 'periodontal disease' OR 'periodontal diseases' OR 'periodontal lesion'):ti,ab,kw)<br>AND<br>('tooth pulp disease'/exp OR 'endodontics'/exp OR (endodontic* OR 'pulp necrosis' OR 'pulp disease' OR 'root canal'):ti,ab,kw)<br>)<br>)<br>AND<br>(<br>'prognosis'/exp OR 'treatment outcome'/exp OR 'tooth loss'/exp OR (prognos* OR predict* OR 'risk factor' OR 'risk factors' OR outcome* OR success* OR failure* OR surviv* OR 'tooth retention' OR extract* OR 'treatment response'):ti,ab,kw<br>)	840

Database	Search strategy (keywords, controlled vocabulary, and Boolean operators)	Records identified (n)
**Web of Science**	(<br>TS=("endodontic-periodontal" OR "endodontic periodontal" OR "periodontal-endodontic" OR "endo-perio" OR endoperiodontal OR "combined endodontic and periodontal" OR "combined periodontal and endodontic")<br>OR<br>(<br>TS=(periodontitis OR "periodontal disease" OR "periodontal diseases" OR "periodontal lesion")<br>AND<br>TS=(endodontic* OR "dental pulp disease" OR "pulp necrosis" OR "pulp disease" OR "root canal")<br>)<br>)<br>AND<br>TS=(prognos* OR predict* OR "risk factor" OR "risk factors" OR outcome* OR success* OR failure* OR surviv* OR "tooth retention" OR extract* OR "treatment response")	586
**Cochrane Library**	#1 MeSH descriptor: [Periodontitis] explode all trees<br>#2 (periodontitis OR "periodontal disease" OR "periodontal diseases" OR "periodontal lesion"):ti,ab,kw<br>#3 #1 OR #2<br>#4 MeSH descriptor: [Dental Pulp Diseases] explode all trees<br>#5 MeSH descriptor: [Endodontics] explode all trees<br>#6 MeSH descriptor: [Root Canal Therapy] explode all trees<br>#7 (endodontic* OR "pulp necrosis" OR "pulp disease" OR "root canal"):ti,ab,kw<br>#8 #4 OR #5 OR #6 OR #7<br>#9 ("endodontic-periodontal" OR "endodontic periodontal" OR "periodontal-endodontic" OR "endo-perio" OR endoperiodontal OR "combined endodontic and periodontal" OR "combined periodontal and endodontic"):ti,ab,kw<br>#10 #9 OR (#3 AND #8)<br>#11 MeSH descriptor: [Prognosis] explode all trees<br>#12 MeSH descriptor: [Treatment Outcome] explode all trees<br>#13 MeSH descriptor: [Tooth Loss] explode all trees<br>#14 (prognos* OR predict* OR "risk factor" OR "risk factors" OR outcome* OR success* OR failure* OR surviv* OR "tooth retention" OR extract* OR "treatment response"):ti,ab,kw<br>#15 #11 OR #12 OR #13 OR #14<br>#16 #10 AND #15	17

Abbreviations: MeSH, Medical Subject Headings; ti, title; ab, abstract; kw, keyword; TS, Topic Search.
